# Effect of Sonication Time on Magnetorheological Effect for Monomodal Magnetic Elastomers

**DOI:** 10.3390/gels4020049

**Published:** 2018-05-23

**Authors:** Mayuko Watanabe, Junko Ikeda, Yoshihiro Takeda, Mika Kawai, Tetsu Mitsumata

**Affiliations:** 1 Graduate School of Science and Technology, Niigata University, Niigata 950-2181, Japan; f18b022j@mail.cc.niigata-u.ac.jp (M.W.); mikagoro@eng.niigata-u.ac.jp (M.K.); 2 ALCA, Japan Science and Technology Agency, Tokyo 102-0076, Japan; 3 Nihon Rufuto Corporation, Tokyo 110-0015, Japan; j.ikeda@nihon-rufuto.com; 4 Rigaku Corporation, Tokyo 196-8666, Japan; y-takeda@rigaku.co.jp

**Keywords:** stimuli-responsive material, magnetic elastomer, magnetic gel, viscoelastic property, magnetorheology, sonication

## Abstract

The effect of sonication time on the storage modulus and particle morphology for magnetic elastomers was investigated by dynamic viscoelastic measurements and morphological studies. An ultrasonic wave using a homogenizer was irradiated to magnetic liquids containing 70 wt % carbonyl iron, for up to 30 min before cure. SEM photographs revealed that magnetic particles were randomly dispersed in the polyurethane matrix for magnetic elastomers with sonication. A parameter showing nonlinear viscoelasticity for magnetic elastomers with sonication decreased from 0.75 to 0.4, indicating that the aggregations of magnetic particles had been destroyed by the sonication. The storage modulus at 500 mT at the linear viscoelastic regime significantly increased with the irradiation time, reaching saturation after 10 min; this suggests an increase in the number of chains of magnetic particles by sonication, due to the random dispersion of magnetic particles. At high strains, the storage modulus at 500 mT increased by 8.9 kPa by sonication, indicating the number of chains of magnetic particles which were not destroyed by increased sonication. It was also found that the storage modulus for polyurethane elastomers without magnetic particles was not varied by sonication, suggesting that the polyurethane network was not broken. The effect of sonication time on the viscoelastic properties, and on the magnetorheological response for magnetic elastomers, is discussed.

## 1. Introduction

Magnetic elastomers are soft materials that are responsive to magnetic fields, and that consist of polymeric matrixes and magnetic particles. When magnetic fields are applied to a magnetic elastomer, the viscoelastic properties alter in response [1,2,3,4]. This is called the magnetorheological effect. So far, we have developed a new class of magnetic soft materials that exhibit drastic and reversible changes in the dynamic modulus by weak magnetic fields [5,6,7]. In addition to the viscoelastic property, the magnetic-field response of physical properties such as surface property [8,9], electric conductivity [10,11], thermal conductivity [12,13,14], have also been reported.

It is well known that the dispersibility of magnetic particles strongly influences the magnetic-field response. The dispersibility of magnetic particles can be improved on a micro- or nano-scale, by the surface modification of magnetic particles [15,16,17,18], resulting in the giant magnetorheological response. For example, a magnetic elastomer with nanoscale homogeneity shows a large increase in Young’s modulus at relatively weak magnetic fields [19]. On the other hand, sonication using an ultrasonic homogenizer is also widely used to improve the dispersibility of inorganic fillers. In fact, there is a substantial amount of literature describing the effect of ultrasonication on the physical properties of polymer composites [20,21,22,23,24,25]. However, surprisingly, there are few reports dealing with the effect of sonication time on magnetorheology, not only for magnetic elastomers, but also for magnetic fluids. In the field of polymer composites for example, Lam et al. reported that there is an optimum sonication time for nanoclay/epoxy composites, whereby mechanical properties are mostly enhanced [23]. Kabir et al. reported that the sonication time for carbon nanofibers and polyurethane composites must be adequately controlled in order to achieve maximum mechanical performance [24]. Cheng et al. reported that an increased sonication time increases the dispersion of single walled carbon nanotubes (SWNTs) in organic solvents [25]. These papers tell us that an optimal condition of sonication exists, also for magnetic elastomers. In this paper, we present the rheological data and morphological data for magnetic elastomers with sonication, and discuss the effect of the irradiation time on these properties and their magnetorheological effects.

## 2. Results and Discussion

**Figure 1:**
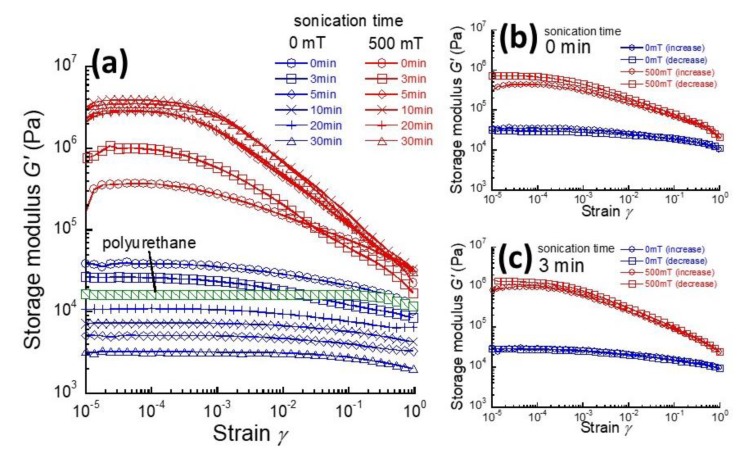
(**a**) Strain dependence of storage modulus for magnetic elastomers with various sonication times at 0 and 500 mT. Hysteresis in storage modulus for magnetic elastomers with sonication: irradiation time (**b**) 0 min and (**c**) 3 min.

Figure 1 shows the strain dependence of the storage modulus for magnetic elastomers, with various sonication times, at 0 and 500 mT. The storage modulus for polyurethane elastomers was almost independent of the strain. The storage modulus for magnetic elastomers at 0 mT gradually decreased with the strain, indicating that the contact among magnetic particles is destroyed by strains. On the other hand, the storage modulus at 500 mT significantly decreased at high strains. This suggests that the chain structure of magnetic particles is destroyed by strains. An interesting phenomenon was observed for magnetic elastomers without sonication at 500 mT (Figure 1b,c): there was a hysteresis in the storage modulus with increasing and decreasing the strain, suggesting the presence of strain-induced alignment of magnetic particles. Magnetic elastomers with sonication did not show any such hysteresis on the storage modulus against strain. At 0 mT, there was no hysteresis on the storage modulus for all magnetic elastomers. These results strongly indicate that magnetic particles in magnetic elastomers with sonication align in the direction of the magnetic field.

**Figure 2:**
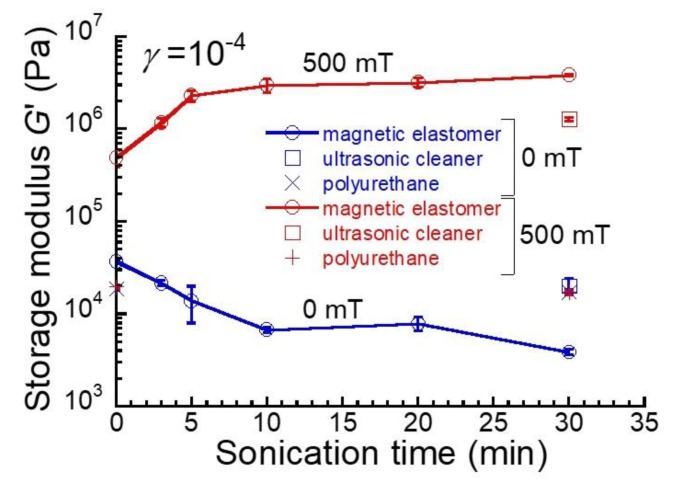
Relationship between storage modulus for magnetic elastomers at *γ* = 10^−4^ and sonication time at 0 mT and 500 mT.

Figure 2 shows the relationship between the storage modulus for magnetic elastomers at *γ* =10^−4^ and the irradiation time of sonication. The storage modulus for magnetic elastomers at 0 mT decreased with the irradiation time. In particular, a large decrease in the storage modulus was seen in the first 10 min. The storage modulus for magnetic elastomers at 500 mT increased markedly in the first 10 min, and reached a constant value after that. This shows that the number of chains increases with sonication. The storage modulus for polyurethane elastomers without sonication was 18.8 ± 0.8 kPa, which was not affected by the sonication (4 plots of ×, + in Figure 2). This indicates that the decrease in the storage modulus is not due to the breakage of polyurethane networks by sonication. It is not yet clear, however, whether a soft phase might have appeared in the magnetic elastomers as a result of sonication.

**Figure 3:**
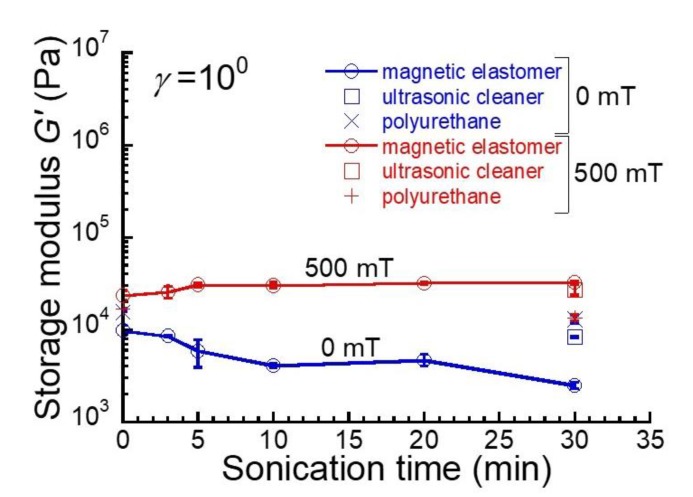
Relationship between storage modulus for magnetic elastomers at *γ* = 1 and sonication time at 0 mT and 500 mT.

Figure 3 shows the relationship between the storage modulus for magnetic elastomers at *γ* = 1 and the irradiation time of sonication. The storage modulus for magnetic elastomers at 0 mT decreased with irradiation time. At 0 mT, the storage modulus for the magnetic elastomer that was sonicated by an ultrasonic cleaner for 30 min was almost the same as that without sonication. On the other hand, the storage modulus at 500 mT increased in the first 5 min, then remained stable at a constant value, suggesting that a relatively large number of chains occurred in large strains for magnetic elastomers that had been sonicated for longer than 5 min. In addition, there was no clear change in the storage modulus for magnetic elastomers with an ultrasonic cleaner and high-power sonication for 30 min. Similar to Figure 2, the storage modulus for polyurethane elastomers was not affected by the magnetic field, independently of the sonication time (4 plots of ×, + in Figure 3).

**Figure 4:**
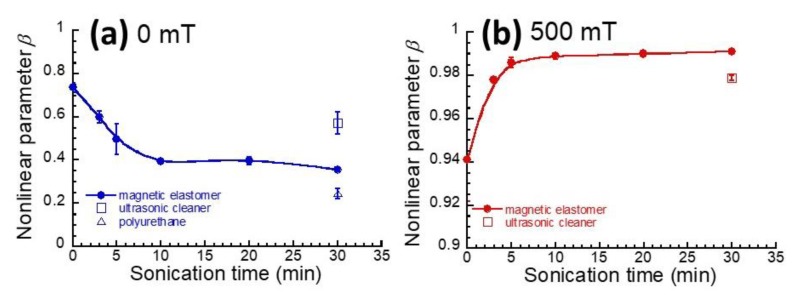
Relationship between nonlinear parameter defined by Equation (1) and sonication time for magnetic elastomers, (**a**) 0 mT, (**b**) 500 mT.

Figure 4a shows the relationship between nonlinear parameter *β* at 0 mT and the irradiation time of sonication for magnetic elastomers. The nonlinear parameter *β* was defined as the following equation [6,8],

(1) $$\beta \equiv 1-\frac{G\prime \left(\gamma =1\right)}{G\prime \left(\gamma =10^{-4}\right)}$$

where *G*′(*γ* = 1) and *G*′(*γ* = 10^−4^) is the storage modulus at strains of 1 and 10^−4^, respectively. The value of *β* decreased significantly with the irradiation time of sonication in the first 10 min, indicating that the dispersibility of magnetic particles was improved by sonication. After 10 min, the *β* showed a constant value of approximately 0.36, which is close to a value of *β* for a polyurethane elastomer without magnetic particles (=0.25). A small, albeit insignificant effect by sonication but was also observed for magnetic elastomers for which sonication was carried out using an ultrasonic cleaner. Figure 4b shows the relationship between nonlinear parameter *β* at 500 mT and the irradiation time of sonication for magnetic elastomers. The value of *β* significantly increased with the irradiation time of sonication in the first 10 min. This indicates that the level of contact between magnetic particles in the chains increased with sonication. After 10 min, the *β* showed a constant value, suggesting that the number of chains does not increase further, even when the sonication is carried out for longer than 10 min. It was also found that the *β* took a high value for sonication using an ultrasonic cleaner compared to that without sonication, indicating that the number of chains increased by sonication.

**Figure 5:**
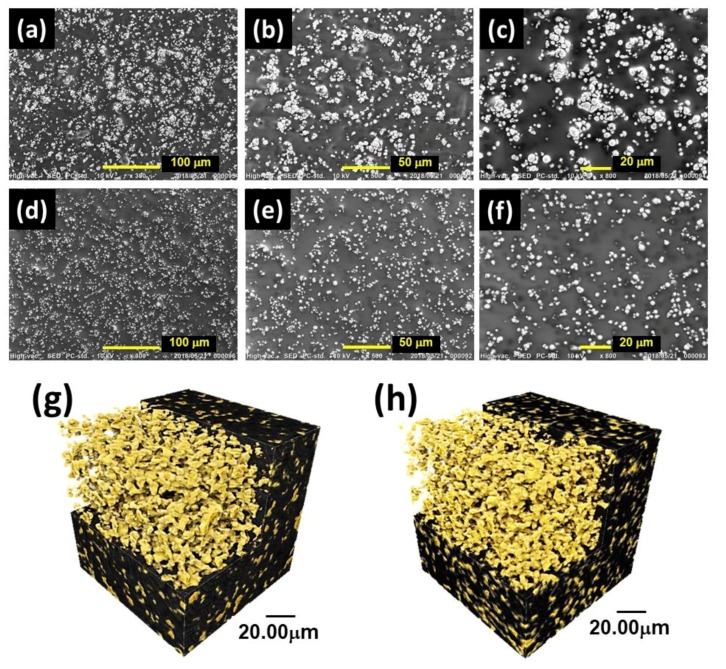
(**a**–**f**) SEM photographs and (**g**,**h**) computed tomography images for magnetic elastomers (**a**–**c**,**g**) without and (**d**–**f**,**h**) with sonication for 20 min.

Figure 5a–f show the SEM photographs for magnetic elastomers containing magnetic particles 70 wt %, without and with sonication (20 min). Agglomerates of magnetic particles were found for magnetic elastomers without sonication. On the other hand, magnetic elastomers with sonication had few aggregations of magnetic particles. Thus, the dispersibility of magnetic particles can be improved by sonication. Figure 5g,h shows the computed tomography (CT) images for magnetic elastomers containing magnetic particles 70 wt %, without and with sonication (20 min). As shown in the SEM photographs, fine magnetic particles were observed in CT photographs.

## 3. Conclusions

The effect of sonication time on the dispersibility of magnetic particles was investigated for magnetic elastomers by rheological measurements and morphological observations. Nonlinear viscoelasticity, SEM, and CT photographs in the absence of a magnetic field revealed that magnetic particles were randomly dispersed in the polyurethane matrix, with less contact among them, by the sonication, resulting in an enhanced magnetorheological response. This strongly indicates that the number of chains of magnetic particles forming under magnetic fields increased by sonication. Due to this, a relatively large number of chains at large strains occurred. We consider that sonication is an effective way to improve the magnetorheological effect at high strains. Additionally, we found that the storage modulus in the absence of a magnetic field decreased with the irradiation time of sonication. This might be an indication that a soft phase appeared through the random dispersion of magnetic particles due to the sonication.

## 4. Materials and Methods

### 4.1. Synthesis of Magnetic Elastomer

**Figure 6:**
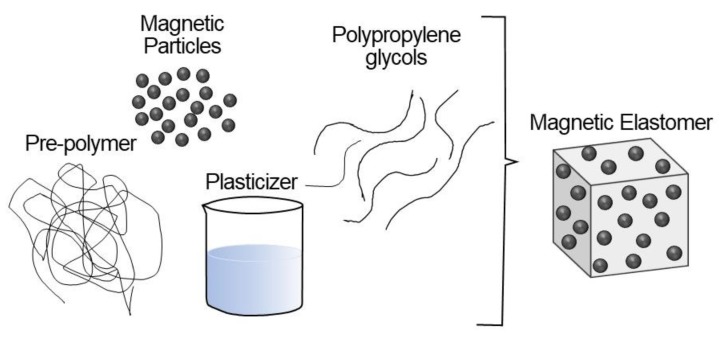
Schematic illustration showing the sample preparation for magnetic elastomers.

Figure 6 shows the schematic illustration for the sample preparation of magnetic elastomers. Polyurethane elastomers and magnetic elastomers were synthesized by a prepolymer method. Polypropylene glycols (*M*w = 2000, 3000), prepolymer cross-linked by tolyrene diisocyanate (Wako Pure Chemical Industries. Ltd., Osaka, Japan), dioctyl phthalate (DOP, Wako Pure Chemical Industries. Ltd.), and carbonyl iron (CS Grade BASF SE., Ludwigshafen am Rhein, Germany) particles were mixed by a mechanical mixer for several minutes. The molar ratio of –NCO to –OH group for the prepolymer was constant, at 2.01 (=[NCO]/[OH]). The median diameter of carbonyl iron particles was 7.0 ± 0.2 μm, determined by a particle size analyzer (SALD-2200, Shimadzu Co., Ltd., Kyoto, Japan). The saturation magnetization for carbonyl iron particles was evaluated to be 245 emu/g, by a SQUID magnetometer (MPMS, Quantum Design Inc., San Diego, CA, USA). Sonication was carried out for 0–30 min using an ultrasonic homogenizer (UD-211, Tomy Seiko Co., Ltd., Tokyo, Japan). The frequency was 20 kHz and the output power 100 W. As a comparison, an ultrasonic cleaner (USM, SND Co., Ltd., Nagano, Japan), which is commercially used as a cleaner for laboratory glasses, was used for this experiment. The frequency is 42 kHz and the output power is 30 W. The mixed liquid was poured into a silicon mold and cured on a hot plate for 20 min at 100 °C. The weight concentration of DOP to the matrix without magnetic particles was fixed at 60 wt %. The weight fraction of the magnetic particles was kept at 70 wt %, which corresponds to a volume fraction of 0.23.

### 4.2. Rheological Measurements

The strain dependence of dynamic modulus was carried out using a rheometer (MCR301, Anton Paar Pty. Ltd., Graz, Austria) at 20 °C. The frequency was constant at 1 Hz. An electric current of 3 A was used to generate a magnetic field of 500 mT. The sample was a disk with a 20 mm diameter and 1.5 mm thickness.

### 4.3. SEM Observations

Scanning electron microscope (SEM) observations were carried out using a JCM-6000 Neoscope (JEOL Ltd., Tokyo, Japan) with an accelerating voltage of 10 kV, without Au coating.

### 4.4. CT Scan Observations

Computed tomography (CT) scan observations were carried out using an X-ray microscope (nano3DX, Rigaku Co., Tokyo, Japan) under no magnetic field. The sample of the magnetic elastomer was cut into a cube with dimensions of 1.0 mm × 1.0 mm × 1.0 mm, and was embedded in an epoxy resin (3.0 mm in diameter, 3.0 mm in height). The weight fraction of the magnetic particles was kept at 70 wt %, which corresponds to a volume fraction of 0.23.
